# Low expression of INMT is associated with poor prognosis but favorable immunotherapy response in lung adenocarcinoma

**DOI:** 10.3389/fgene.2022.946848

**Published:** 2022-11-10

**Authors:** Xincheng Zhou, Bing Zou, Jian Wang, Lihong Wu, Qiang Tan, Chunyu Ji

**Affiliations:** ^1^ Department of Experimental Pathology, Ningbo Clinical Pathological Diagnosis Center, Ningbo, China; ^2^ Department of Radiation Oncology, Cancer Hospital of Shandong Province, Jinan, China; ^3^ Burning Rock Biotech, Guangzhou, China; ^4^ Shanghai Lung Tumor Clinical Medical Center, Shanghai Chest Hospital, Shanghai Jiao Tong University, Shanghai, China; ^5^ Department of Thoracic Surgery, Shanghai Chest Hospital, Shanghai Jiao Tong University, Shanghai, China

**Keywords:** INMT, lung adenocarcinoma, prognosis, immunotherapy, cell cycle

## Abstract

**Background:** The expression of INMT (indolethylamine N-methyltransferase) has been reported to be downregulated in non-small-cell lung cancer (NSCLC). However, the role of INMT in NSCLC remains elusive. We aim to investigate the underlying mechanisms and clinical value of INMT in NSCLC, especially in lung adenocarcinoma (LUAD).

**Methods:** Gene expression cohorts from The Cancer Genome Atlas (TCGA) and Gene Expression Omnibus (GEO) were analyzed to assess the effect of INMT on NSCLC. Gene expression data from an immunotherapy cohort were used to investigate the association of INMT with immunotherapy in NSCLC.

**Results:** INMT expression was significantly downregulated in NSCLC compared with adjacent normal tissues. Downregulated INMT was associated with poor overall survival in LUAD, but not in lung squamous carcinoma. Multivariate Cox regression analysis suggested that INMT was an independent prognostic marker in LUAD. INMT had a reference value in the diagnosis and prognostic estimation of LUAD. Gene set enrichment analysis showed that pathways of the cell cycle and DNA damage response were enriched in the INMT low-expression group. The top 10 hub genes upregulated in the INMT low-expression group mainly activated the cell cycle pathway. In addition, more frequently mutated *TP53* genes, higher aneuploidy scores, a fraction of genomes altered, MANTIS scores, and tumor mutation burden were found in tumors with low expression of INMT. Furthermore, patients with low expression of INMT showed favorable clinical benefits to anti-PD-1 treatment with higher enrichment scores of immune-related signatures.

**Conclusion:** The low expression of INMT was associated with poor prognosis but favorable immunotherapy response in LUAD. INMT may affect the progression of LUAD by regulating the cell cycle and may serve as a valuable independent prognostic biomarker in patients with LUAD.

## Introduction

Non-small-cell lung cancer (NSCLC) is a malignant cancer that has the highest mortality rate of all cancers worldwide (Siegel et al., 2020; Sung et al., 2021). Lung adenocarcinoma (LUAD) is the largest subtype of NSCLC (Little et al., 2007). Advances in recent years, such as the identification of multiple oncogenic drivers and the use of immunotherapies, have changed the treatment of LUAD (Kleczko et al., 2019). However, the survival rates remain low. Therefore, it is urgent to find more effective biomarkers to smooth the way for novel therapeutic methods.

In recent years, immunotherapy, especially immune checkpoint inhibitors (ICIs) targeting programmed cell death-1 (PD-1) and its ligand PD-L1, has revolutionized cancer treatment and substantially improved patient outcomes in NSCLC (Suresh et al., 2018). However, only a limited subset of patients could benefit from immunotherapy, and immunotherapy lacks precise biomarkers to predict efficacy (Brahmer et al., 2012). Therefore, identifying biomarkers to screen dominant populations for ICI efficacy is particularly important. Multiple factors associated with the clinical outcome of immunotherapy are discovered, such as PD-L1 expression (Herbst et al., 2014; Shukuya and Carbone, 2016), tumor mutation burden (TMB) (Rizvi et al., 2015), DNA mismatch repair deficiency (Le et al., 2015), the degree of cytotoxic T-cell infiltration (Tang et al., 2016), mutational signature (Miao et al., 2018), antigen presentation defects (Chowell et al., 2018), interferon signaling (Ayers et al., 2017), and tumor aneuploidy (Davoli et al., 2017). These biomarkers show different accuracies and utilities, and identifying robust ICI-response biomarkers remains a critical challenge in the field (Nishino et al., 2017).

Indolethylamine N-methyltransferase (INMT) is a methyltransferase that regulates the tryptophan metabolic pathway by catalyzing the N-methylation of tryptamine and structurally related compounds (Chu et al., 2014; Torres et al., 2019). As a thioether S-methyltransferase, it also plays an important role in the detoxification of selenium compounds (Kuehnelt et al., 2015). It is specifically expressed in the lung and expressed as supplemental in the liver, kidneys, prostate, and other tissues (Fukumoto et al., 2020). It has been reported that the expression of INMT is downregulated in lung cancer, prostate cancer, and meningioma (Kopantzev et al., 2008; Larkin et al., 2012; Schulten et al., 2016). However, the role of INMT and its molecular mechanism in cancer, especially lung cancer, remain unknown. The study of the molecular mechanism of INMT would help us better understand the process of tumorigenesis and development and find new targets in cancers. Herein, using data from The Cancer Genome Atlas (TCGA) project and the Gene Expression Omnibus (GEO) database, we performed a secondary analysis to thoroughly analyze the INMT expression level, determine its prognostic role, and explore its potential functions in NSCLC.

## Materials and methods

### Genomic data sources

The transcriptome sequencing data (including 962 NSCLC samples and 103 adjacent nontumor samples), somatic mutation data (including 486 LUAD samples), and clinical information of TCGA data were downloaded from the Genomic Data Commons (GDC) data portal (https://portal.gdc.cancer.gov/). The samples from primary lesions that had a follow-up time of more than 1 month were included in this study. The following gene expression profiles were downloaded from GEO (www.ncbi.nlm.nih.gov/geo/): GSE19188 (including 65 tumor samples and 72 adjacent nontumor samples), GSE72094 (including 398 LUAD samples), and GSE41271 (including 183 LUAD samples); these were used to further validate our results. The PD-1 immunotherapy gene expression profiling dataset GSE135222 (including 27 NSCLC samples) was downloaded from GEO and used to analyze the association between INMT expression and immunotherapy response. The detailed data sources used in this study are summarized in Supplementary Table S1.

### Establishment and evaluation of the nomogram for lung adenocarcinoma survival prediction

In this study, all independent prognostic factors were selected using multivariate Cox regression analysis and used to construct the nomogram to evaluate the 3- and 5-year overall survival (OS) probabilities of LUAD patients. Covariates in the nomogram were assessed for the patient and given a point. A higher total number of points represented a lower expected survival. By comparing the predicted probability of the line chart with the observed actual probability through a calibration curve, the accuracy of the line chart was verified. The overlapping reference lines show that the model is accurate.

### Differential gene expression analysis

The “limma” package (version 3.46.0), using R software, was used to screen differentially expressed genes (DEGs) between INMT low- and high-expression groups. INMT-related DEGs were identified when the adjusted *p*-value < 0.05 and |log_2_(Fold Change) | > 1.

### Gene set enrichment analysis and functional annotation

Gene set enrichment analysis (GSEA) was performed to explore the biological functions of INMT in LUAD (Subramanian et al., 2005). First, we ranked all the mRNAs according to the fold change between INMT high- and low-expression groups. Then, the ordered mRNAs were imported to the R package “clusterProfiler” (version 3.18.1) for GSEA, containing KEGG and Reactome pathways from a Molecular Signatures Database (MSigDB) (https://software.broadinstitute.org/gsea/msigdb). Benjamini–Hochberg standard false discovery rate correction was used for multiple testing corrections. The gene set was considered significantly enriched when the adjusted *p*-value < 0.05.

### Protein–protein interaction network construction and hub gene identification

The protein–protein interaction (PPI) data were extracted from the Search Tool for the Retrieval of Interacting Genes (STRING) database (https://string-db.org/), an online tool allowing users to upload the data of DEGs. It is used to analyze the PPI information and to evaluate the interaction relationships among DEGs (Szklarczyk et al., 2015). After downloading INMT-related DEG interactions, the PPI network was visualized using Cytoscape (3.7.2) software (http://www.cytoscape.org/). In Cytoscape, module screening and connection degree computation were performed using the maximal clique centrality (MCC) method in the cytoHubba plugin. Nodes with a higher degree of connection were more essential for maintaining the stability of the entire network; usually, nodes with a degree of connection ≥10 were considered to be core candidate genes. In this study, the top 10 hub genes were selected for further functional analysis. The GeneMANIA database (http://www.genemania.org) was also applied to construct the INMT interaction network.

### The relationship between gene expression and pathway activity in GSCALite

Gene Set Cancer Analysis (GSCALite) (http://bioinfo.life.hust.edu.cn/web/GSCALite/) is a web-based platform for dynamic analysis and visualization of gene sets from the point of view of the expression of malignant tumor genes correlations with drug sensitivity (Liu et al., 2018). The correlation between gene expression and pathway activity groups (activation and inhibition) defined by pathway scores was analyzed in GSCALite. Pathway activation (red) represents the percentage of cancers in which the pathway may be activated by given genes, and inhibition in a similar way is shown as pathway inhibition (blue).

### The relationship between gene expression and drug sensitivity in GSCALite

The drug sensitivity analysis of GSCALite has collected 481 small molecules from the Cancer Therapeutics Response Portal (CTRP) (https://portals.broadinstitute.org/ctrp/) (Rees et al., 2016). Drug sensitivity and gene expression profiling data on cancer cell lines in CTRP are integrated for investigation (Garnett et al., 2012). The expression profiling of each gene in a given gene set is performed by Spearman’s correlation analysis with small molecule/drug sensitivity (IC_50_). The Spearman correlation represents the gene expression that correlates with the drug. A negative correlation means that the gene’s high expression is sensitive to the drug and *vice versa*.

### Mutational analysis

The R package “maftools” (version 2.6.05) was used to analyze the frequently mutated genes in the TCGA-LUAD cohort. Aneuploidy scores, a fraction of genome altered, MANTIS scores, and TMB scores were downloaded from cBioPortal (https://www.cbioportal.org/study/clinicalData?id=luad_tcga_pan_can_atlas_2018). TMB scores of the immunotherapy cohort were downloaded from GEO (https://www.ncbi.nlm.nih.gov/geo/query/acc.cgi?acc=GSE135222). The aneuploidy score is the total number of arm-level gains and losses for a tumor, adjusted for ploidy. The fraction of genome altered is the percentage of copy number-altered chromosome regions out of measured regions. The MANTIS score is a score that predicts a patient’s microsatellite instability (MSI) status (Bonneville et al., 2017). TMB is broadly defined as the number of nonsynonymous somatic mutations per megabase of the interrogated genomic sequence as previously described (Chalmers et al., 2017).

### Immune gene signature calculation

Immuno-Oncology Biological Research (IOBR) is a tool for leveraging multi-omics data to facilitate immuno-oncology exploration and unveil tumor–immune interactions (Zeng et al., 2021). The gene sets utilized for the immune signature score in this study are defined as previously reported (Ayers et al., 2017; Charoentong et al., 2017; Mariathasan et al., 2018) and are presented in Supplementary Table S2. The enrichment scores of these immune gene signatures were calculated using the “IOBR” R package (version 0.99.9).

### Statistical analysis

The Student *t*-test or Wilcoxon rank-sum test was used to compare two groups of continuous variables, depending on whether the data were normally distributed. The Chi-squared test or Fisher’s exact test was used to compare categorical variables. The Spearman correlation test was applied to evaluate the correlation between sample factors. Receiver operating characteristic (ROC) analysis was performed to assess the diagnostic value of INMT expression in NSCLC. The Kaplan–Meier method was applied for survival analysis, and the log-rank test was used to estimate statistical significance. Multivariate Cox regression analysis was used to screen potential prognostic factors. The level of significance was set at *p* < 0.05, and all statistical tests were two-sided. All statistical data analyses were implemented using R software, version 4.0.2.

## Results

### Indolethylamine N-methyltransferase was significantly downregulated in patients with NSCLC

**FIGURE 1:**
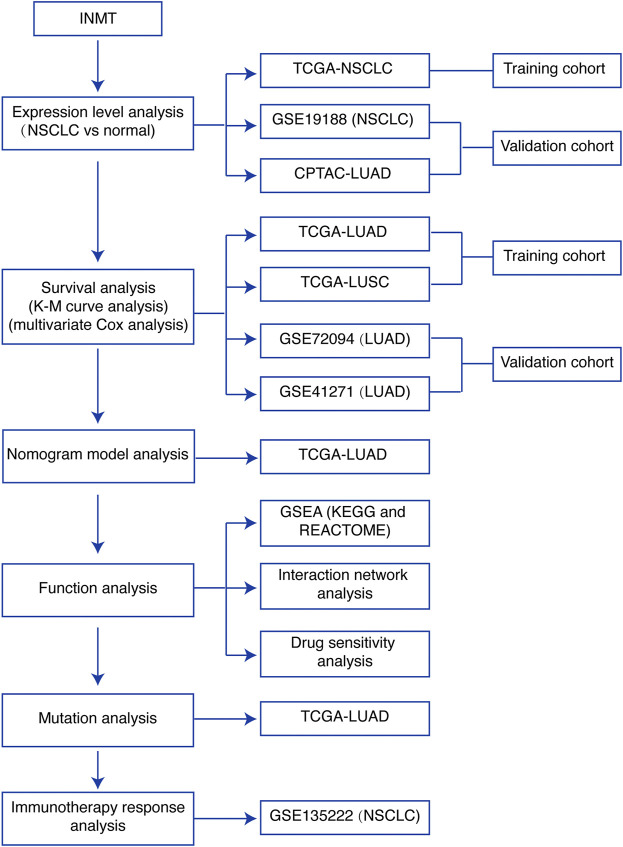
Study flowchart. INMT, indolethylamine N-methyltransferase; NSCLC, non-small-cell lung cancer; LUAD, lung adenocarcinoma; LUSC, lung squamous cell carcinoma; TCGA, The Cancer Genome Atlas; K-M curve, Kaplan–Meier survival; GSEA, gene set enrichment analysis.

**FIGURE 2:**
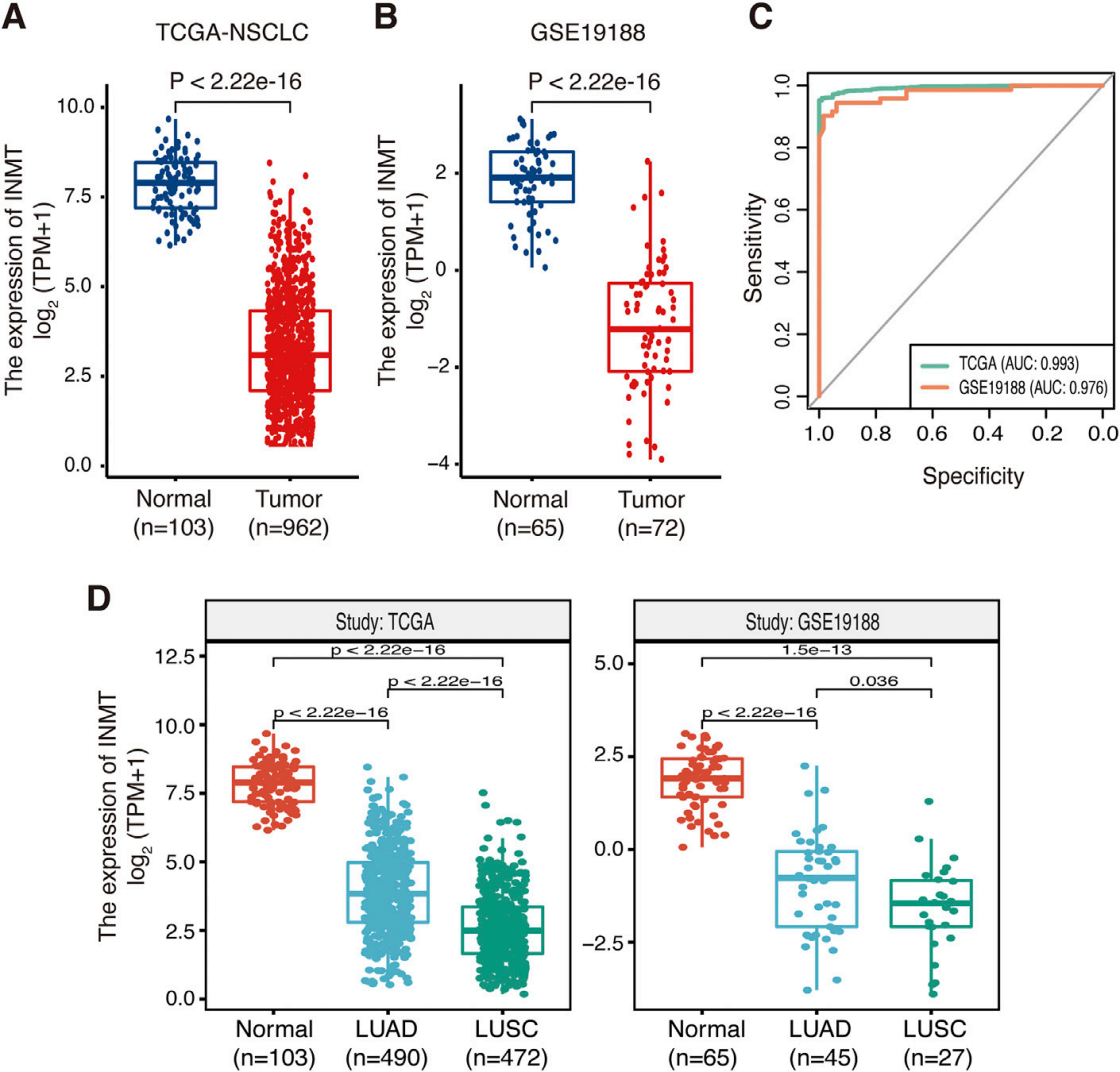
Expression of INMT in normal lung and NSCLC tissues. **(A,B)** Differential expression of INMT in normal lung and NSCLC tissues in TCGA dataset **(A)** and the GSE19188 dataset **(B)**. **(C)** Evaluation of the sensitivity and specificity of NSCLC diagnosis by ROC curves in TCGA dataset and the GSE19188 dataset. **(D)** Differential expression of INMT in normal lung, LUAD, and LUSC tissues in TCGA dataset (left) and the GSE19188 dataset (right), respectively. NSCLC, non-small-cell lung cancer; LUAD, lung adenocarcinoma; LUSC, lung squamous cell carcinoma; ROC, receiver operator characteristic; AUC, area under the curve; TPM, transcripts per million mapped reads.

A brief flowchart of our study is shown in Figure 1. We first used the TCGA-NSCLC database to evaluate the mRNA expression levels of INMT in NSCLC patients and adjacent normal tissues. The result showed that the expression level of INMT in NSCLC was significantly lower than that in normal tissues (*p* < 0.001) (Figure 2A). This result was verified in GSE19188 and CPTAC-LUAD cohorts at a transcription level and protein level, respectively (Figure 2B and Supplementary Figure S1A). According to ROC curve analysis, INMT was a robust predictor of NSCLC, with an area under the curve (AUC) = 0.976 (Figure 2C). Furthermore, we found that the expression of INMT in LUSC was significantly lower than that in LUAD (Figure 2D). Additionally, based on the TCGA cohort, we used the Spearman rank correlation test to analyze the correlation of INMT expression with a pathological stage in LUAD and LUSC. We observed a weak but significant negative correlation between INMT expression and pathological stage in both LUAD and LUSC, i.e., INMT expression decreases as the stage increases (Supplementary Figures S1B, S1C). These results showed that INMT was significantly downregulated in patients with NSCLC and was a robust predictor of NSCLC.

### Low indolethylamine N-methyltransferase expression is associated with poor prognosis of patients with lung adenocarcinoma

**FIGURE 3:**
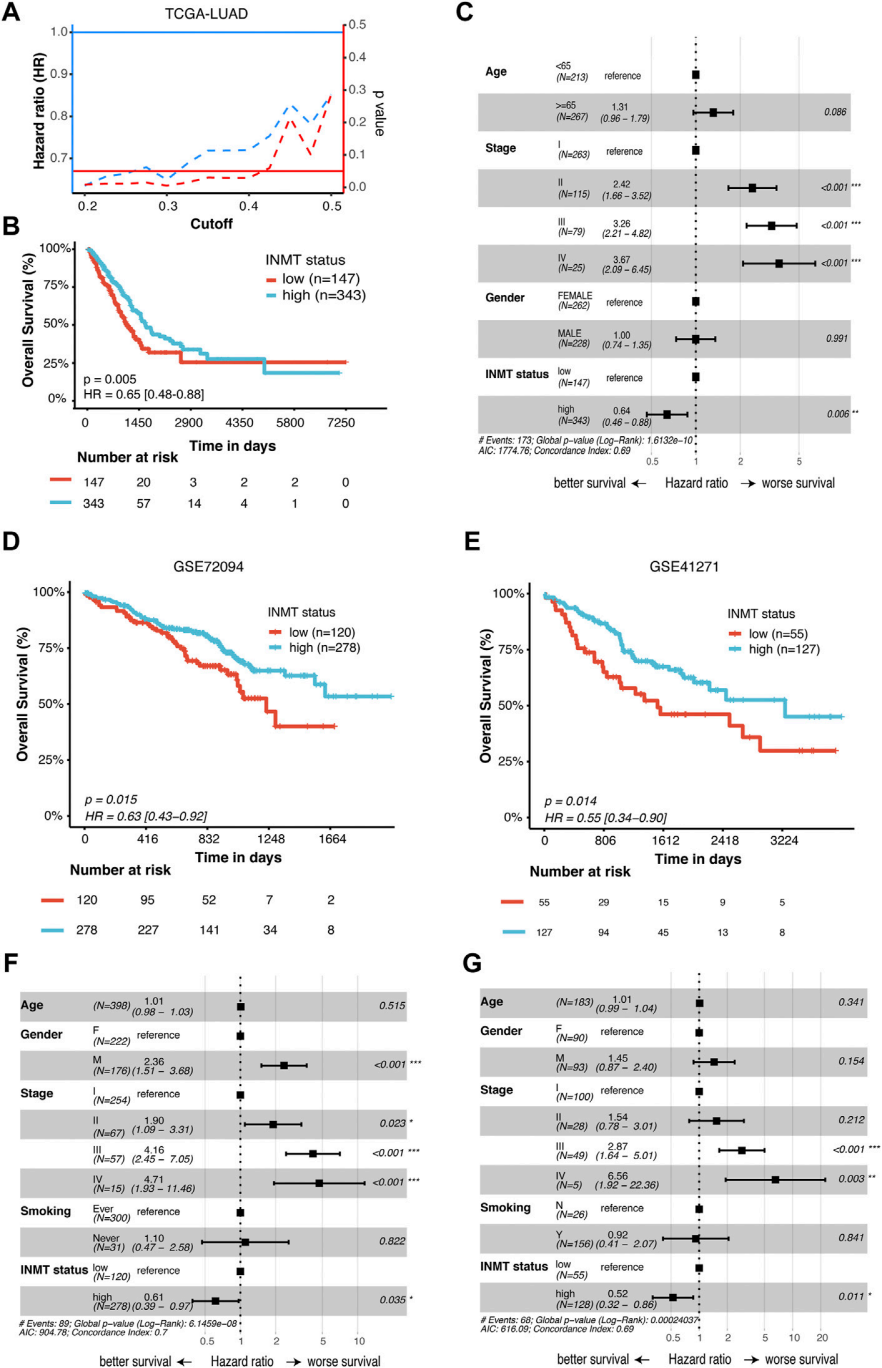
Prognostic significance of INMT in LUAD patients. **(A)** Hazard ratio (HR) and statistical results of the INMT high-expression group *versus* low-expression group at different cutoffs in TCGA-LUAD cohort. The blue dashed line represents the HR value, the red dashed line represents the *p*-value, and the solid red line represents *p* = 0.05. **(B)** Kaplan–Meier curve analysis of the prognostic significance of high- and low-expression of INMT in TCGA-LUAD cohorts. **(C)** Multivariate Cox analysis of the clinical characteristics and INMT associated with overall survival (OS) in TCGA-LUAD cohort. **(D,E)** Kaplan–Meier curve analysis of the prognostic significance of high and low expression of INMT in two GEO-LUAD cohorts (GSE72094 and GSE41271), respectively. **(F,G)** Multivariate Cox analysis of the clinical characteristics and INMT associated with OS in two GEO-LUAD cohorts (GSE72094 and GSE41271), respectively. The cutoff of 30% quantile was used to divide patients into low- and high-expression groups. LUAD, lung adenocarcinoma; HR, hazard ratio; OS, overall survival.

To identify whether INMT expression affects patient survival, Kaplan–Meier survival analysis was conducted on the TCGA-NSCLC cohort. As shown in Figure 3A, low INMT expression was associated with poor prognosis in LUAD patients. At the cutoff value of a quantile of 30%, the survival difference between the low-INMT group and the high-INMT group was the most significant in LUAD patients. So, we used this cutoff to classify LUAD patients into a low INMT expression group (30% of samples with the lowest expression) and a high INMT expression group (the remaining 70% of the samples) in this study. The Kaplan–Meier survival analysis showed that the low expression of INMT was significantly related to the poor OS of LUAD patients [Hazard ratio (HR), 1.54; 95% CI, 1.14-2.08; *p*-value = 0.005] (Figure 3B). Multivariate Cox regression analysis results suggested that INMT expression was an independent prognosis factor in the TCGA-LUAD cohort, after adjusting age, gender, and pathological stage (Figure 3C). Similarly, we also checked the association between INMT expression and survival in LUSC patients. However, the Kaplan–Meier survival analysis failed to show a significant difference between low and high INMT expression groups in LUSC patients (Supplementary Figures S2A, S2B). Furthermore, we validated the relationship between INMT expression and OS using two GEO-LUAD cohorts (GSE72094 and GSE41271) and demonstrated that INMT was an independent prognosis factor in LUAD patients through multivariate Cox analysis (Figures 3D–G).

### Prognostic nomogram model for lung adenocarcinoma overall survival

**FIGURE 4:**
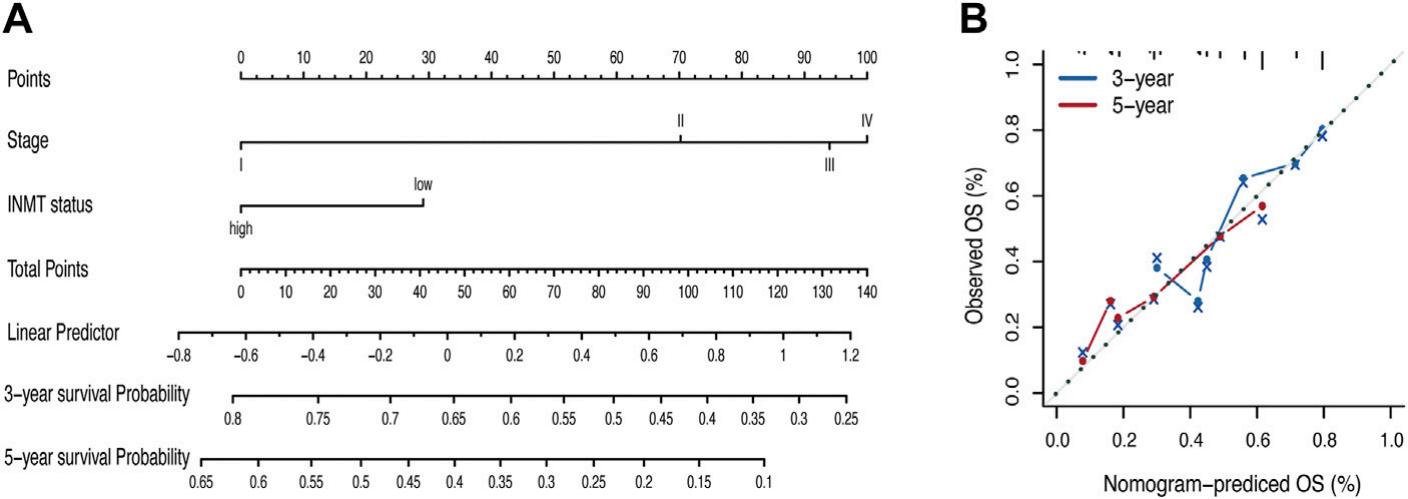
Nomogram for the prediction of survival in LUAD. **(A)** Nomogram by multivariate Cox regression analysis for predicting the proportion of patients with overall survival (OS). **(B)** Plots depict the calibration of the model in terms of the agreement between predicted and observed OS. Model performance is shown by the plot, relative to the 45-degree line, which represents perfect prediction. OS, overall survival.

To better predict the prognosis of LUAD patients in the clinic, we developed a prognostic nomogram model by integrating two independent predictors of mortality from the aforementioned analyses, INMT expression and pathological stage, into a multivariate Cox regression model, which was evaluated and validated using TCGA, GSE72094, and GSE41271 data (Figures 3C, F, G). A score based on the nomogram developed in the current study was calculated to predict the 3- and 5-year survival probabilities for individual patients (Figure 4A). The calibration plot showed that the nomogram performed well in predicting patient OS according to an ideal model (Figure 4B).

### Low indolethylamine N-methyltransferase expression is closely related to the cell cycle, DNA replication, and DNA damage response pathways

**FIGURE 5:**
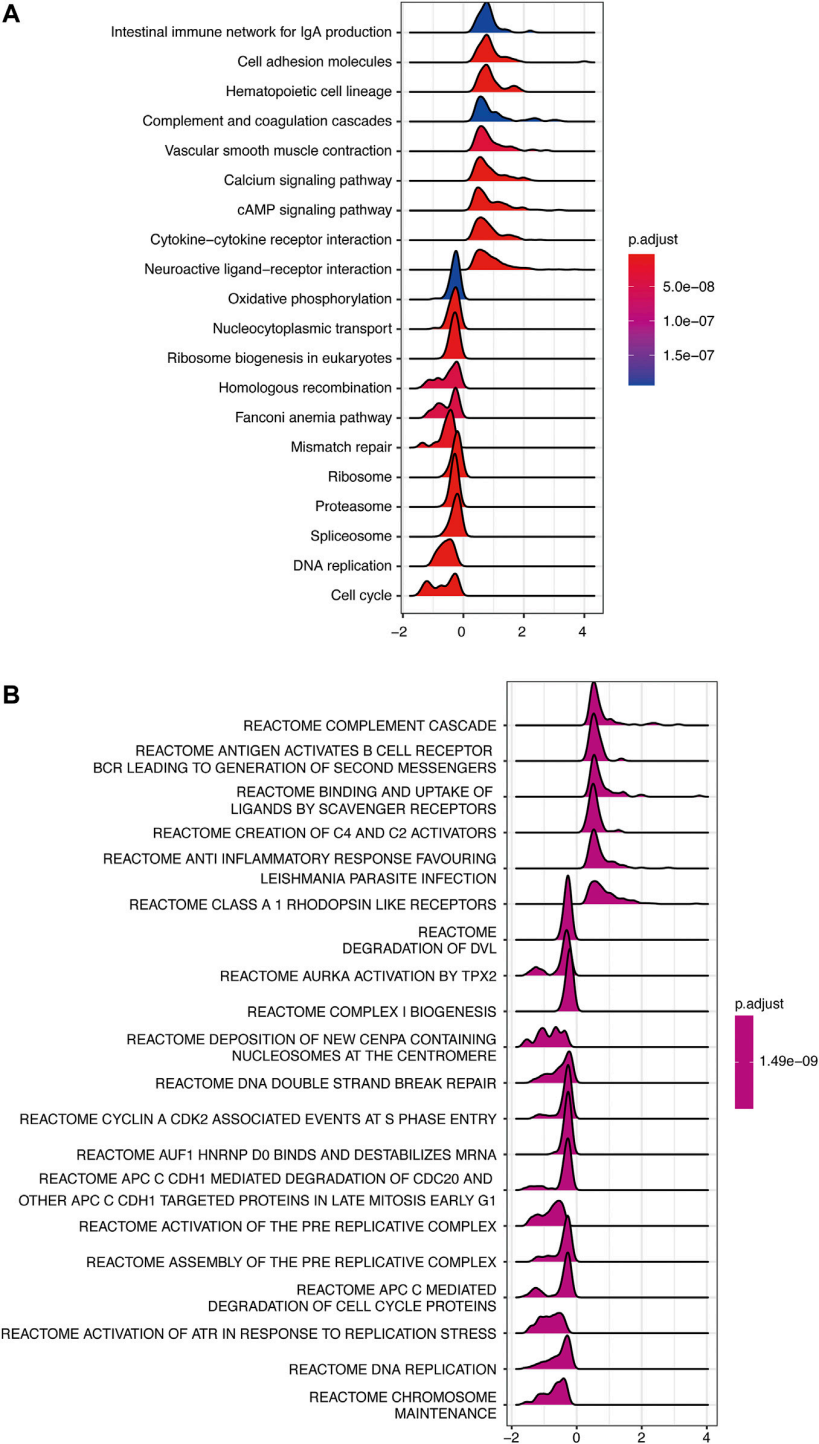
GSEA of samples between high- and low-INMT groups in TCGA-LUAD cohort. Ridge plot of gene sets of KEGG **(A)** and Reactome **(B)** enriched in the high- or low-INMT group in TCGA-LUAD cohort. The X-axis represents the normalized enrichment score (NES), and the color represents the *p*-value adjusted by FDR. The top enriched signaling pathways are shown in the figures. GSEA, gene set enrichment analysis.

To investigate the possible signaling pathways in which INMT might be involved, GSEA was performed on the TCGA-LUAD cohort. Supplementary Tables S3 and S4 illustrate GSEA results of KEGG and Reactome gene sets between high- and low-INMT groups, respectively. As shown in Figure 5A, KEGG gene sets of the cell cycle, DNA replication, and DNA damage response (DDR) pathways, such as mismatch repair, Fanconi anemia pathway, and homologous recombination, were enriched in the INMT low-expression group. GSEA of Reactome gene sets showed similar results that the INMT low-expression group was closely associated with cell cycle, DNA replication, and DDR pathways (Figure 5B).

### Hub genes upregulated in the indolethylamine N-methyltransferase low-expression group are associated with cell cycle, apoptosis, and DDR pathways

**FIGURE 6:**
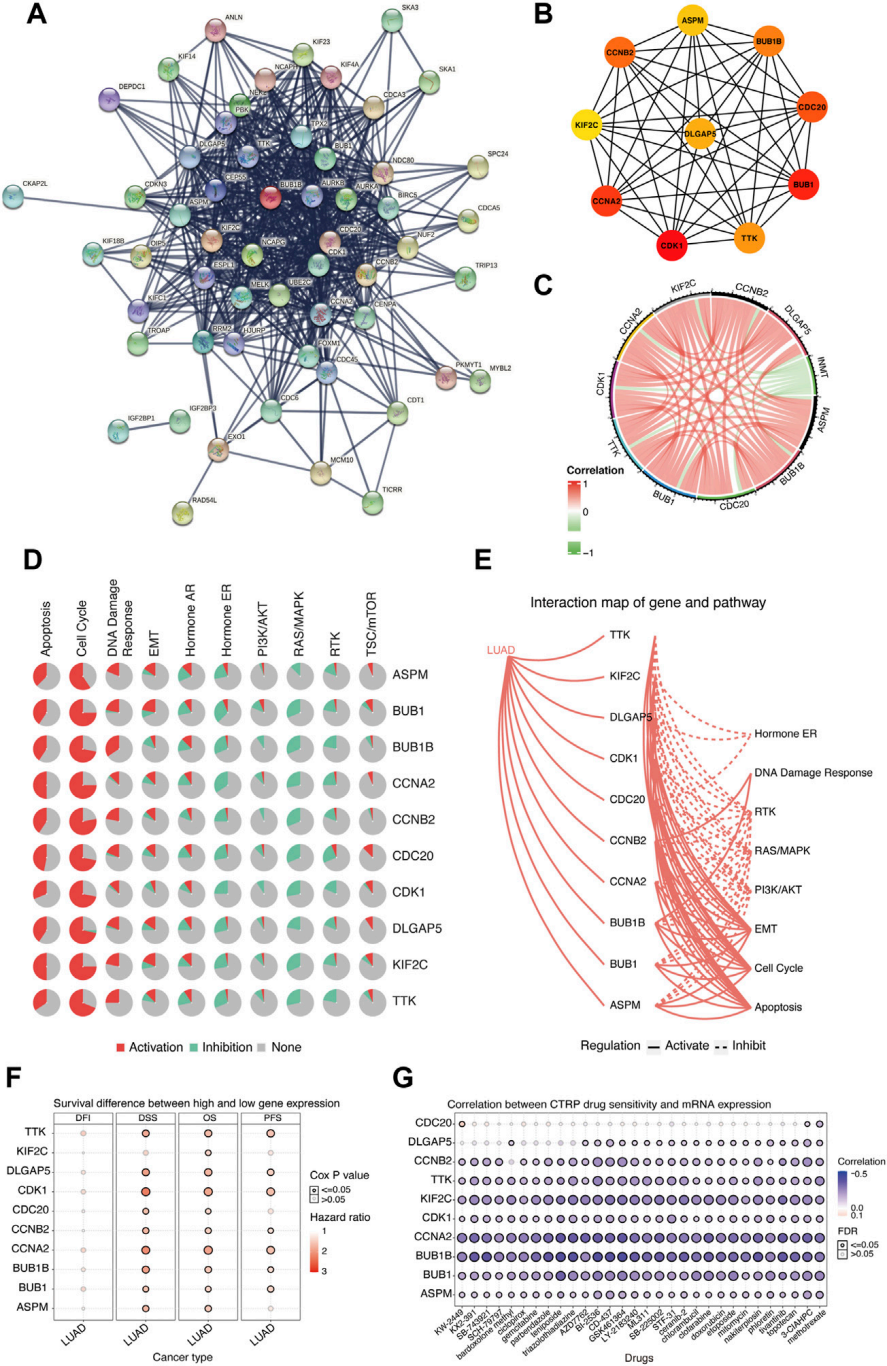
Biological function analysis of hub genes that were upregulated in the INMT low-expression group of TCGA-LUAD cohort. **(A)** Protein–protein interaction (PPI) network of upregulated genes in the INMT low-expression group constructed based on the STRING database. **(B)** Top 10 hub genes identified using the MCC method in the cytoHubba plugin of Cytoscape. **(C)** Chord plot for the correlation of INMT and its hub genes. **(D)** Pathway activity analysis of hub genes. Pathway activation (red) represents the percentage of cancers in which pathways may be activated by given genes, and inhibition in a similar way showed as pathway inhibition (blue). **(E)** Interaction map of hub genes and pathway conducted. A solid line indicates that the hub gene activates the pathway, and a dashed line indicates that the hub gene inhibits the pathway. **(F)** Survival difference between the high and low expression of hub genes. **(G)** Correlation between the expression of hub genes and CTRP drug sensitivity. The analyses of Figures 5D–G were performed online in GSCALite. LUAD, lung adenocarcinoma; PPI, protein–protein interaction.

Considering that downregulated INMT was associated with the worse prognosis in LUAD, we further explored the functions of hub genes that were upregulated in the INMT low-expression group of the TCGA-LUAD cohort to find the potential drugs that were inhibitors of hub genes for these INMT-related high-risk patients. As shown in Figure 6A, 111 upregulated genes in the INMT low-expression group were used to construct a PPI network based on the STRING database and thus formed 56 nodes and 553 edges. The top 10 hub genes were identified from these complex interactomes using the MCC method in Cytoscape, namely, ASPM, BUB1, BUB1B, TTK, CDC20, CDK1, CCNA2, CCNB2, DLGAP5, and KIF2C (Figure 6B). The chord plot result confirmed that the expression of each hub gene was negatively correlated with the expression of INMT (Figure 6C). Furthermore, pathway activity analysis of hub genes indicated that the pathways of the cell cycle, apoptosis, and DDR, the vital steps in tumor progression, were mainly activated by these 10 hub genes (Figures 6D,E). In addition, high expression of each hub gene was significantly associated with a worse OS in LUAD (Figure 6F), which was consistent with the association of low INMT with poor OS. Additionally, we used CTRP IC_50_ drug data from the GSCALite database to analyze the correlation between the expression of these 10 hub genes and the sensitivity of the small-molecule drugs in LUAD cell lines. We found that LUAD cell lines with hub gene overexpression were sensitive to the cell cycle and DNA replication-related drugs, such as topotecan, etoposide, doxorubicin, and gemcitabine (Figure 6G). Our aforementioned results found that these hub genes upregulated in the INMT low-expression group were mainly involved in the activation of the cell cycle, apoptosis, and DDR pathways and might provide the basis for drug-targeted therapy for these INMT-related high-risk LUAD patients.

### Low indolethylamine N-methyltransferase indicates high-frequency somatic alterations

**FIGURE 7:**
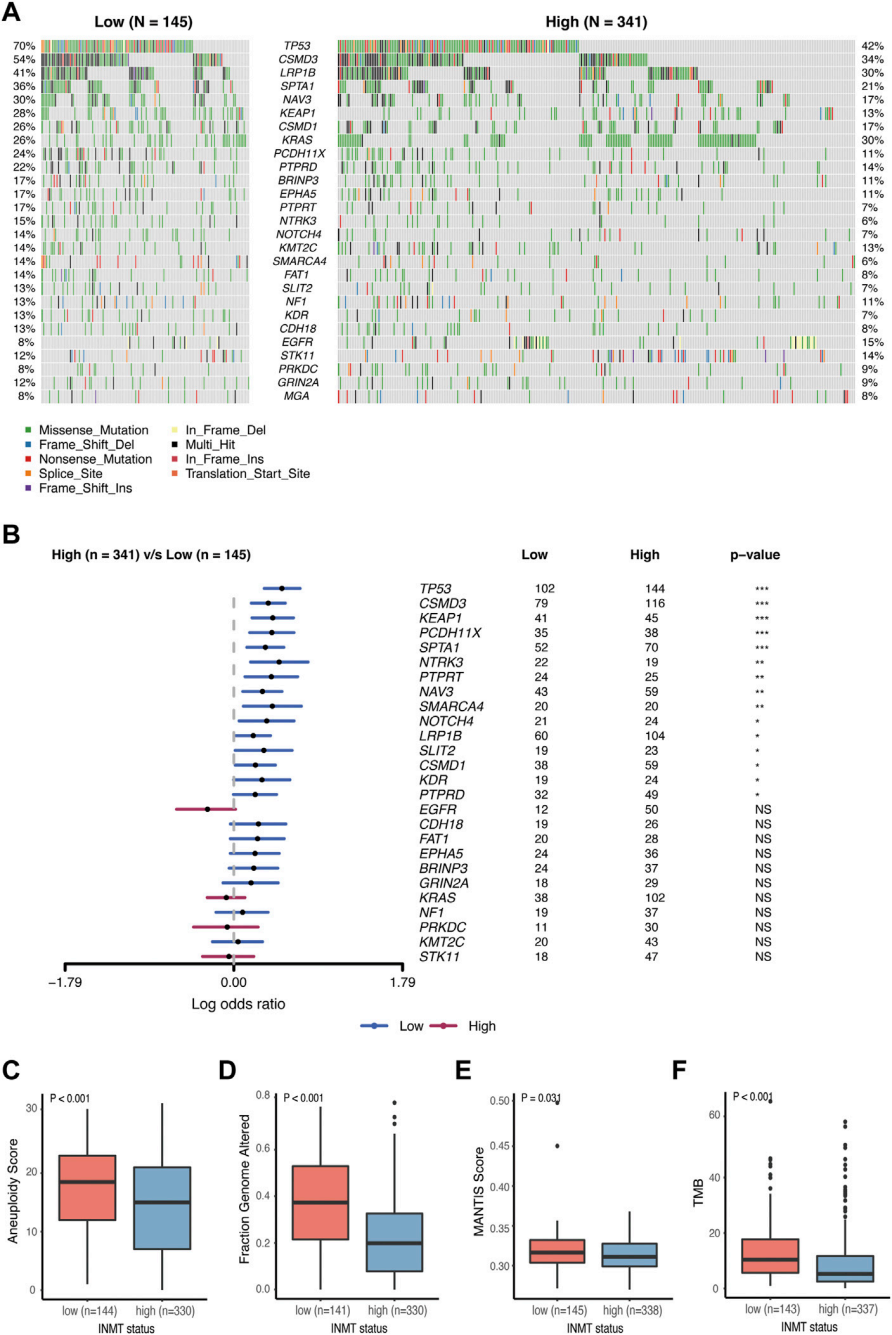
Association between INMT and gene alterations in TCGA-LUAD cohort. **(A)** Common tumor-related gene mutation information illustrated in the somatic mutation spectrum in low- and high-INMT groups, respectively. The genes in the top 20 of the population mutation frequency are shown in the figure. **(B)** Forest plot examined the difference in the population frequency of mutant genes between the high- and low-INMT groups. **(C–F)** Distribution of the aneuploidy score **(C)**, a fraction of genome altered score **(D)**, MANTIS score **(E)**, and TMB score **(F)** between low- and high-INMT groups. TMB, tumor mutation burden.

It has been reported that somatic mutations were involved in the development of cancer (Martincorena and Campbell, 2015). Here, we used the TCGA-LUAD cohort to investigate the difference in somatic mutations between low- and high-INMT groups in LUAD. Common tumor-related mutations were shown in the waterfall plot and stratified by the INMT expression level (Figure 7A). Somatic mutation profiles revealed that the tumor-suppressor gene TP53 was more frequently mutated in the low INMT expression group (Figure 7B). We then compared the differences in the distribution of aneuploidy scores, a fraction of genome altered scores, MANTIS scores, and TMB scores between low- and high-INMT groups. We found that the low INMT expression group had higher aneuploidy scores, a fraction of genome altered scores, MANTIS scores, and TMB scores (Figures 7C–F).

### Low indolethylamine N-methyltransferase is associated with a favorable immunotherapy response

**FIGURE 8:**
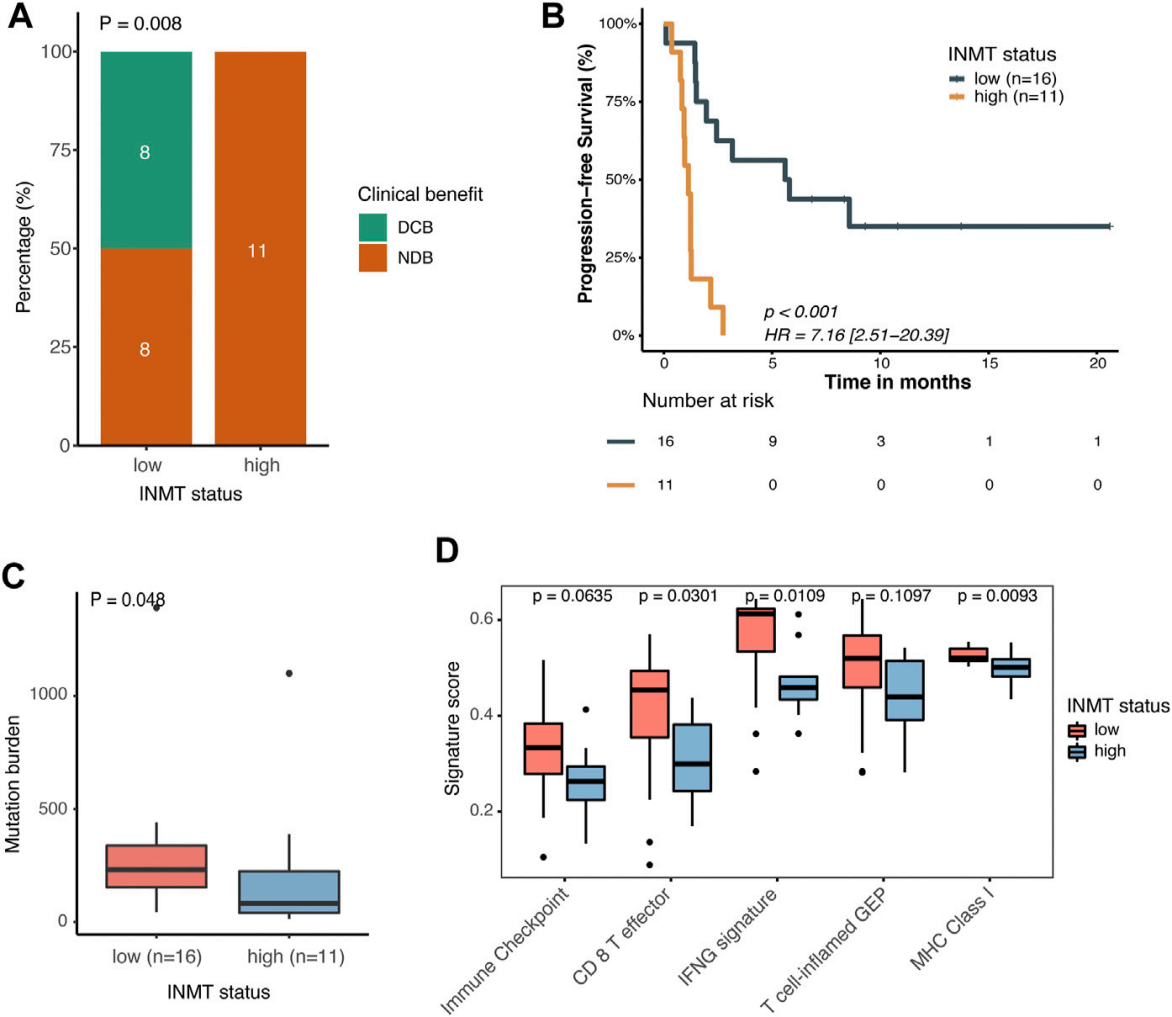
Association between INMT and immunotherapy response in a PD-1 immunotherapy cohort of NSCLC (GSE135222). **(A)** Clinical benefit rate among INMT low-and high-expression groups. Fisher’s exact test was used for the analysis. **(B)** Kaplan–Meier curve for progression-free survival according to an INMT expression status. The log-rank test was used for the analysis. **(C)** TMB distribution between INMT low- and high-expression groups. The Wilcoxon rank-sum test was used for the analysis. **(D)** Boxplot of enrichment scores of immune-related signatures among INMT low- and high-expression groups. The Wilcoxon rank-sum test was used for the analysis. DCB, durable clinical benefit; NDB, non-durable clinical benefit; HR, hazard ratio; TMB, tumor mutation burden.

We previously found that mutated *TP53* genes and higher TMB and MANTIS scores were enriched in tumors with the low expression of INMT, and we therefore speculated on whether these high-risk patients would benefit from immunotherapy. We then investigated the correlation between INMT expression and immunotherapy response in a GEO public PD-1 immunotherapy cohort of advanced NSCLC (GSE135222). As shown in Figures 8A, B, patients with low INMT expression had a higher durable clinical benefit (DCB) rate (50% vs. 0%, *p* = 0.008) and more improved progression-free survival (PFS) (HR, 0.14; 95% CI, 0.05–0.40; *p* < 0.001) than those with high INMT expression, with median PFS of 5.70 months vs. 1.73 months. We also checked the distribution of TMB in low-and high-INMT groups and found that tumors with low INMT expression had higher TMB (Figure 8C). Previous studies have confirmed that CD8 effector T cells, MHC Class I, IFN-gamma signaling, and T-cell-inflamed gene expression profiling (GEP) play roles in anticancer immunity and immunotherapeutic effects (Ayers et al., 2017; Charoentong et al., 2017; Mariathasan et al., 2018). Here, we analyzed the relationship between INMT and these immune signatures and found that the signature scores of CD8 effector T cells, IFN-gamma signaling, and MHC Class I signature were significantly higher in the INMT low-expression group (Figure 8D).

## Discussion

In this study, we used data from TCGA and GEO to thoroughly analyze the INMT expression level, determine its prognostic role, and explore its potential functions in NSCLC. We found that INMT expression was significantly downregulated in NSCLC, and downregulated INMT was associated with poor OS in LUAD, but not in LUSC. Multivariate Cox regression analysis further demonstrated that INMT is a promising independent prognostic biomarker in LUAD in three independent datasets. In addition, INMT has a certain reference value for the diagnosis and prognosis of LUAD. GSEA results found that pathways of the cell cycle, DNA replication, and DDR were enriched in the INMT low-expression group. The top 10 hub genes upregulated in the INMT low-expression group mainly activated the cell cycle pathway, and LUAD cell lines with hub gene overexpression were sensitive to the cell cycle and DNA replication-related drugs. More mutated *TP53* genes and higher aneuploidy scores, a fraction of genome altered scores, MANTIS scores, and TMB scores were found in the INMT low-expression group. Furthermore, a GEO public PD-1 immunotherapy cohort of NSCLC suggested that patients in the INMT-related high-risk group could benefit from immunotherapy. Our study has provided new insights into INMT that could be a potential prognostic marker of survival and a potential predictive marker of immunotherapy in LUAD patients.

As a methyltransferase, INMT detoxifies selenium compounds and regulates the tryptophan metabolic pathway by catalyzing the N-methylation of tryptamines and structure-related compounds (Kuehnelt et al., 2015). INMT is downregulated in NSCLC and prostate cancer (Kopantzev et al., 2008; Larkin et al., 2012; Jianfeng et al., 2022). To the best of our knowledge, no previous study has assessed the relationship between INMT and prognosis in cancers. In this work, using TCGA-NSCLC data, we found that the low expression of INMT was associated with poor OS in LUAD, but not in LUSC. We further demonstrated that INMT was an independent prognostic biomarker in LUAD using multivariate Cox regression analysis in TCGA-LUAD and another two GEO cohorts. We found that INMT expression decreased as the pathological stage increased; this supported that there was a correlation between low INMT and poor prognosis in LUAD. ROC curve analysis and the nomogram model showed that INMT had a certain reference value in the diagnosis and prognostic estimation of LUAD. Our work is the first report on the association between INMT and the prognosis of patients with LUAD, providing new insights into INMT as a potential prognostic marker in LUAD.

Previous studies on INMT have mainly focused on its role in regulating the tryptophan metabolic pathway and detoxifying selenium compounds by catalyzing the methylation of several substrates (Chu et al., 2014; Kuehnelt et al., 2015; Torres et al., 2019). Only a few studies have reported on its biological function in prostate cancer but not in lung cancer. For instance, Zhong et al. (2021) found that INMT was highly increased in castration-resistant prostate cancer, and further *in vitro* experiments suggested that INMT might promote prostate cancer castration resistance through detoxification of anticancer metabolites. Wang et al. found that INMT may inhibit proliferation and promote apoptosis of human prostate cancer cells (Jianfeng et al., 2022). In our study, we showed that the cell cycle and DNA replication pathways were enriched in the INMT low-expression group. Cell cycle disorder is one of the key features of cancer that cause genomic instability (Hanahan and Weinberg, 2011). Our GSEA results indicated that downregulated INMT may lead to an acceleration of cell cycle and DNA replication to increase the probability of genome instability. Further hub gene analysis also showed that the top 10 hub genes that were upregulated in the INMT low-expression group played key roles in the control of the cell cycle, including mitotic spindle regulation and G1/S and G2/M transition. The drug sensitivity analysis revealed that LUAD cell lines with hub gene overexpression were sensitive to the cell cycle and DNA replication-related drugs, indicating that these INMT-related high-risk patients might benefit from cell cycle-related drugs. Our results revealed that INMT may affect the progression of LUAD by regulating the cell cycle and might provide the basis for drug-targeted therapy for these INMT-related high-risk LUAD patients. However, our results are only analyzed based on the public data, and further molecular experiments on cancer cell lines are needed to explore the mechanism of INMT in tumorigenesis and the development of LUAD.

Our analysis of the relationship between INMT expression and immunotherapy response found that NSCLC patients with low INMT expression showed favorable clinical benefits to anti-PD-1 treatment. More mutated TP53 genes, higher TMB, and higher enrichment scores of immune-related signatures of MHC Class I, CD8^+^ effector T cells, and IFN-gamma signaling were found in the INMT low-expression group. A TP53 gene mutation has been reported to boost PD-L1 expression, facilitate T-cell infiltration, and augment tumor immunogenicity and is a potential predictive marker for response to ICIs in LUAD (Dong et al., 2017). TMB reflects cancer mutation quantity. The more mutations there are, the higher the number of neoantigens and the higher the chances that one or more of the neoantigens will be immunogenic and trigger a T-cell response. Many studies have reported a connection between higher TMB and ICI efficacy across a wide variety of cancer types (Snyder et al., 2014; Goodman et al., 2017; Cristescu et al., 2018; Samstein et al., 2019). A number of predicted MHC Class I-associated neoantigens have been shown to be correlated with a cytolytic activity (Rooney et al., 2015), and the anti-tumor activity of ICIs is dependent on MHC Class I presentation of specific tumor-derived peptides (Gubin et al., 2014; Tran et al., 2015). CD8^+^ T cells are primed and activated toward CD8^+^ T effector cells in a process called the cancer immunity cycle to make durable and efficient anti-tumor immune responses (Chen and Mellman, 2013). It has been reported that the IFN-γ–related mRNA profile could predict clinical response to a PD-1 blockade in many types of cancers (Ayers et al., 2017). The higher scores of biomarkers in the INMT low-expression group may explain why patients in the INMT low-expression group have a better response to immunotherapy in NSCLC. Although our result is based on a small cohort, it provides these INMT-related high-risk patients with a treatment option.

There are several limitations to our work. First, we did not investigate the exact mechanisms of INMT with *in vivo/in vitro* experiments, and further experiments are required to demonstrate the effect of INMT on the tumor cell cycle to improve the reliability of our results. Second, we obtained data on the anti-PD-1 response in a small NSCLC cohort from a public database, and further immunotherapy data on LUAD are needed to verify the role of INMT.

## Conclusion

In summary, INMT is downregulated in LUAD, and the low expression of INMT is closely associated with poor prognosis in LUAD. INMT has a certain reference value for the diagnosis and prognosis of LUAD. Furthermore, INMT may affect the progression of LUAD by regulating the cell cycle. With further exploration, patients with low INMT expression showed favorable clinical benefits to anti-PD-1 treatment. This is the first study to reveal that INMT influences prognosis and immunotherapy responses in LUAD. These findings provide a new perspective on LUAD progression and treatment.

## Data Availability

The original contributions presented in the study are included in the article/Supplementary Material; further inquiries can be directed at the corresponding authors.

## Abbreviations

AUC, area under the curve; CTRP, Cancer Therapeutics Response Portal; DCB, durable clinical benefit; DDR, DNA damage response; DEGs, differentially expressed genes; GDC, Genomic Data Commons; GEO, Gene Expression Omnibus; GEP, gene expression profiling; GSEA, gene set enrichment analysis; HR, hazard ratio; ICIs, immune checkpoint inhibitors; INMT, indolethylamine N-methyltransferase; IOBR, Immuno-Oncology Biological Research; LUAD, lung adenocarcinoma; LUSC, lung squamous cell carcinoma; MCC, maximal clique centrality; MSI, microsatellite instability; MSigDB, Molecular Signatures Database; NSCLC, non-small-cell lung cancer; OS, overall survival; PD-1, programmed cell death-1; PFS, progression-free survival; PPI, protein–protein interaction; ROC, receiver operating characteristic; STRING, Search Tool for Retrieval of Interacting Genes; TCGA, The Cancer Genome Atlas; TMB, tumor mutation burden; TPM, transcripts per million mapped reads.

## References

[B1] Ayers M. Lunceford J. Nebozhyn M. Murphy E. Loboda A. Kaufman D. R. (2017). IFN-gamma-related mRNA profile predicts clinical response to PD-1 blockade. J. Clin. Invest.. 127 (8), 2930–2940. 10.1172/JCI91190 28650338 PMC5531419

[B2] Bonneville R. Krook M. A. Kautto E. A. Miya J. Wing M. R. Chen H. Z. (2017). Landscape of microsatellite instability across 39 cancer types. JCO Precis. Oncol. 2017, 1–15. 10.1200/PO.17.00073 PMC5972025 29850653

[B3] Brahmer J. R. Tykodi S. S. Chow L. Q. M. Hwu W. J. Topalian S. L. Hwu P. (2012). Safety and activity of anti-PD-L1 antibody in patients with advanced cancer. N. Engl. J. Med. 366 (26), 2455–2465. 10.1056/NEJMoa1200694 22658128 PMC3563263

[B4] Chalmers Z. R. Connelly C. F. Fabrizio D. Gay L. Ali S. M. Ennis R. (2017). Analysis of 100, 000 human cancer genomes reveals the landscape of tumor mutational burden. Genome Med. 9 (1), 34. 10.1186/s13073-017-0424-2 28420421 PMC5395719

[B5] Charoentong P. Finotello F. Angelova M. Mayer C. Efremova M. Rieder D. (2017). Pan-cancer immunogenomic analyses reveal genotype-immunophenotype relationships and predictors of response to checkpoint blockade. Cell Rep. 18 (1), 248–262. 10.1016/j.celrep.2016.12.019 28052254

[B6] Chen D. S. Mellman I. (2013). Oncology meets immunology: The cancer-immunity cycle. Immunity 39 (1), 1–10. 10.1016/j.immuni.2013.07.012 23890059

[B7] Chowell D. Morris L. G. T. Grigg C. M. Weber J. K. Samstein R. M. Makarov V. (2018). Patient HLA class I genotype influences cancer response to checkpoint blockade immunotherapy. Science 359 (6375), 582–587. 10.1126/science.aao4572 29217585 PMC6057471

[B8] Chu U. B. Vorperian S. K. Satyshur K. Eickstaedt K. Cozzi N. V. Mavlyutov T. (2014). Noncompetitive inhibition of indolethylamine-N-methyltransferase by N, N-dimethyltryptamine and N, N-dimethylaminopropyltryptamine. Biochemistry 53 (18), 2956–2965. 10.1021/bi500175p 24730580 PMC4025572

[B9] Cristescu R. Mogg R. Ayers M. Albright A. Murphy E. Yearley J. (2018). Pan-tumor genomic biomarkers for PD-1 checkpoint blockade-based immunotherapy. Science 362 (6411), eaar3593. 10.1126/science.aar3593 30309915 PMC6718162

[B10] Davoli T. Uno H. Wooten E. C. Elledge S. J. (2017). Tumor aneuploidy correlates with markers of immune evasion and with reduced response to immunotherapy. Science 355 (6322), eaaf8399. 10.1126/science.aaf8399 28104840 PMC5592794

[B11] Dong Z. Y. Zhong W. Z. Zhang X. C. Su J. Xie Z. Liu S. Y. (2017). Potential predictive value of TP53 and KRAS mutation status for response to PD-1 blockade immunotherapy in lung adenocarcinoma. Clin. Cancer Res. 23 (12), 3012–3024. 10.1158/1078-0432.CCR-16-2554 28039262

[B12] Fukumoto Y. Yamada H. Matsuhashi K. Okada W. Tanaka Y. K. Suzuki N. (2020). Production of a urinary selenium metabolite, trimethylselenonium, by thiopurine S-methyltransferase and indolethylamine N-methyltransferase. Chem. Res. Toxicol. 33 (9), 2467–2474. 10.1021/acs.chemrestox.0c00254 32786394

[B13] Garnett M. J. Edelman E. J. Heidorn S. J. Greenman C. D. Dastur A. Lau K. W. (2012). Systematic identification of genomic markers of drug sensitivity in cancer cells. Nature 483 (7391), 570–575. 10.1038/nature11005 22460902 PMC3349233

[B14] Goodman A. M. Kato S. Bazhenova L. Patel S. P. Frampton G. M. Miller V. (2017). Tumor mutational burden as an independent predictor of response to immunotherapy in diverse cancers. Mol. Cancer Ther. 16 (11), 2598–2608. 10.1158/1535-7163.MCT-17-0386 28835386 PMC5670009

[B15] Gubin M. M. Zhang X. Schuster H. Caron E. Ward J. P. Noguchi T. (2014). Checkpoint blockade cancer immunotherapy targets tumour-specific mutant antigens. Nature 515 (7528), 577–581. 10.1038/nature13988 25428507 PMC4279952

[B16] Hanahan D. Weinberg R. A. (2011). Hallmarks of cancer: The next generation. Cell 144 (5), 646–674. 10.1016/j.cell.2011.02.013 21376230

[B17] Herbst R. S. Soria J. C. Kowanetz M. Fine G. D. Hamid O. Gordon M. S. (2014). Predictive correlates of response to the anti-PD-L1 antibody MPDL3280A in cancer patients. Nature 515 (7528), 563–567. 10.1038/nature14011 25428504 PMC4836193

[B18] Jianfeng W. Yutao W. Jianbin B. (2022). Indolethylamine-N-Methyltransferase inhibits proliferation and promotes apoptosis of human prostate cancer cells: A mechanistic exploration. Front. Cell Dev. Biol. 10, 805402. 10.3389/fcell.2022.805402 35252179 PMC8891133

[B19] Kleczko E. K. Kwak J. W. Schenk E. L. Nemenoff R. A. (2019). Targeting the complement pathway as a therapeutic strategy in lung cancer. Front. Immunol. 10, 954. 10.3389/fimmu.2019.00954 31134065 PMC6522855

[B20] Kopantzev E. P. Monastyrskaya G. S. Vinogradova T. V. Zinovyeva M. V. Kostina M. B. Filyukova O. B. (2008). Differences in gene expression levels between early and later stages of human lung development are opposite to those between normal lung tissue and non-small lung cell carcinoma. Lung Cancer 62 (1), 23–34. 10.1016/j.lungcan.2008.02.011 18394749

[B21] Kuehnelt D. Engstrom K. Skroder H. Kokarnig S. Schlebusch C. Kippler M. (2015). Selenium metabolism to the trimethylselenonium ion (TMSe) varies markedly because of polymorphisms in the indolethylamine N-methyltransferase gene. Am. J. Clin. Nutr. 102 (6), 1406–1415. 10.3945/ajcn.115.114157 26537946

[B22] Larkin S. E. HolmeS S. Cree I. A. Walker T. Basketter V. Bickers B. (2012). Identification of markers of prostate cancer progression using candidate gene expression. Br. J. Cancer 106 (1), 157–165. 10.1038/bjc.2011.490 22075945 PMC3251845

[B23] Le D. T. Uram J. N. Wang H. Bartlett B. R. Kemberling H. Eyring A. D. (2015). PD-1 blockade in tumors with mismatch-repair deficiency. N. Engl. J. Med. 372 (26), 2509–2520. 10.1056/NEJMoa1500596 26028255 PMC4481136

[B24] Little A. G. Gay E. G. Gaspar L. E. Stewart A. K. (2007). National survey of non-small cell lung cancer in the United States: Epidemiology, pathology and patterns of care. Lung Cancer 57 (3), 253–260. 10.1016/j.lungcan.2007.03.012 17451842

[B25] Liu C. J. Hu F. F. Xia M. X. Han L. Zhang Q. Guo A. Y. (2018). GSCALite: A web server for gene set cancer analysis. Bioinformatics 34 (21), 3771–3772. 10.1093/bioinformatics/bty411 29790900

[B26] Mariathasan S. Turley S. J. Nickles D. Castiglioni A. Yuen K. Wang Y. (2018). TGFβ attenuates tumour response to PD-L1 blockade by contributing to exclusion of T cells. Nature 554 (7693), 544–548. 10.1038/nature25501 29443960 PMC6028240

[B27] Martincorena I. Campbell P. J. (2015). Somatic mutation in cancer and normal cells. Science 349 (6255), 1483–1489. 10.1126/science.aab4082 26404825

[B28] Miao D. Margolis C. A. Vokes N. I. Liu D. Taylor-Weiner A. Wankowicz S. M. (2018). Genomic correlates of response to immune checkpoint blockade in microsatellite-stable solid tumors. Nat. Genet. 50 (9), 1271–1281. 10.1038/s41588-018-0200-2 30150660 PMC6119118

[B29] Nishino M. Ramaiya N. H. Hatabu H. Hodi F. S. (2017). Monitoring immune-checkpoint blockade: Response evaluation and biomarker development. Nat. Rev. Clin. Oncol. 14 (11), 655–668. 10.1038/nrclinonc.2017.88 28653677 PMC5650537

[B30] Rees M. G. Seashore-Ludlow B. Cheah J. H. Adams D. J. Price E. V. Gill S. (2016). Correlating chemical sensitivity and basal gene expression reveals mechanism of action. Nat. Chem. Biol. 12 (2), 109–116. 10.1038/nchembio.1986 26656090 PMC4718762

[B31] Rizvi N. A. Hellmann M. D. Snyder A. Kvistborg P. Makarov V. Havel J. J. (2015). Cancer immunology. Mutational landscape determines sensitivity to PD-1 blockade in non-small cell lung cancer. Science 348 (6230), 124–128. 10.1126/science.aaa1348 25765070 PMC4993154

[B32] Rooney M. S. Shukla S. A. Wu C. J. Getz G. Hacohen N. (2015). Molecular and genetic properties of tumors associated with local immune cytolytic activity. Cell 160 (1-2), 48–61. 10.1016/j.cell.2014.12.033 25594174 PMC4856474

[B33] Samstein R. M. Lee C. H. Shoushtari A. N. Hellmann M. D. Shen R. Janjigian Y. Y. (2019). Tumor mutational load predicts survival after immunotherapy across multiple cancer types. Nat. Genet. 51 (2), 202–206. 10.1038/s41588-018-0312-8 30643254 PMC6365097

[B34] Schulten H. J. Hussein D. Al-Adwani F. Karim S. Al-Maghrabi J. Al-Sharif M. (2016). Microarray expression data identify DCC as a candidate gene for early meningioma progression. PLoS One 11 (4), e0153681. 10.1371/journal.pone.0153681 27096627 PMC4838307

[B35] Shukuya T. Carbone D. P. (2016). Predictive markers for the efficacy of anti-PD-1/PD-L1 antibodies in lung cancer. J. Thorac. Oncol. 11 (7), 976–988. 10.1016/j.jtho.2016.02.015 26944305 PMC7179759

[B36] Siegel R. L. Miller K. D. Jemal A. (2020). Cancer statistics. Ca. Cancer J. Clin. 70 (1), 7–30. 10.3322/caac.21590 31912902

[B37] Snyder A. Makarov V. Merghoub T. Yuan J. Zaretsky J. M. Desrichard A. (2014). Genetic basis for clinical response to CTLA-4 blockade in melanoma. N. Engl. J. Med. 371 (23), 2189–2199. 10.1056/NEJMoa1406498 25409260 PMC4315319

[B38] Subramanian A. Tamayo P. Mootha V. K. Mukherjee S. Ebert B. L. Gillette M. A. (2005). Gene set enrichment analysis: A knowledge-based approach for interpreting genome-wide expression profiles. Proc. Natl. Acad. Sci. U. S. A. 102 (43), 15545–15550. 10.1073/pnas.0506580102 16199517 PMC1239896

[B39] Sung H. Ferlay J. Siegel R. L. Laversanne M. Soerjomataram I. Jemal A. (2021). Global cancer statistics 2020: GLOBOCAN estimates of incidence and mortality worldwide for 36 cancers in 185 countries. Ca. Cancer J. Clin. 71 (3), 209–249. 10.3322/caac.21660 33538338

[B40] Suresh K. Naidoo J. Lin C. T. Danoff S. (2018). Immune checkpoint immunotherapy for non-small cell lung cancer: Benefits and pulmonary toxicities. Chest 154 (6), 1416–1423. 10.1016/j.chest.2018.08.1048 30189190 PMC6335259

[B41] Szklarczyk D. Franceschini A. Wyder S. Forslund K. Heller D. Huerta-Cepas J. (2015). STRING v10: Protein-protein interaction networks, integrated over the tree of life. Nucleic Acids Res. 43, D447–D452. 10.1093/nar/gku1003 25352553 PMC4383874

[B42] Tang H. Wang Y. Chlewicki L. K. Zhang Y. Guo J. Liang W. (2016). Facilitating T cell infiltration in tumor microenvironment overcomes resistance to PD-L1 blockade. Cancer Cell 29 (3), 285–296. 10.1016/j.ccell.2016.02.004 26977880 PMC4794755

[B43] Torres B. Tyler J. S. Satyshur K. A. Ruoho A. E. (2019). Human indole(ethyl)amine-N-methyltransferase (hINMT) catalyzed methylation of tryptamine, dimethylsulfide and dimethylselenide is enhanced under reducing conditions - a comparison between 254C and 254F, two common hINMT variants. PLoS One 14 (7), e0219664. 10.1371/journal.pone.0219664 31310642 PMC6634407

[B44] Tran E. Ahmadzadeh M. Lu Y. C. Gros A. Turcotte S. Robbins P. F. (2015). Immunogenicity of somatic mutations in human gastrointestinal cancers. Science 350 (6266), 1387–1390. 10.1126/science.aad1253 26516200 PMC7445892

[B45] Zeng D. Ye Z. Shen R. Yu G. Wu J. Xiong Y. (2021). Iobr: Multi-Omics immuno-oncology biological research to decode tumor microenvironment and signatures. Front. Immunol. 12, 687975. 10.3389/fimmu.2021.687975 34276676 PMC8283787

[B46] Zhong S. Jeong J. H. Huang C. Chen X. Dickinson S. I. Dhillon J. (2021). Targeting INMT and interrupting its methylation pathway for the treatment of castration resistant prostate cancer. J. Exp. Clin. Cancer Res. 40 (1), 307. 10.1186/s13046-021-02109-z 34587977 PMC8482636

