# Supplementary material for: Low expression of INMT is associated with poor prognosis but favorable immunotherapy response in lung adenocarcinoma
Source: Front Genet. 2022 Nov 10;13:946848. doi: 10.3389/fgene.2022.946848 (PMC9686007; doi:10.3389/fgene.2022.946848)
Supplement: Supplementary file 1 [file Presentation1.zip › Supplementary Figures & Tables.PDF]

Supplemental materials for

Low expression of INMT is associated with poor prognosis but favorable immunotherapy response in lung adenocarcinoma

Xincheng Zhou[1†], Bing Zou[2†], Jian Wang[3], Lihong Wu[3], Qiang Tan[4*], Chunyu Ji[5*]

*** Correspondence:**

Chunyu Ji, Department of Thoracic Surgery, Shanghai Chest Hospital, Shanghai Jiao Tong University, Shanghai 200030, China.

E-mail: chunyuji2022@163.com

Qiang Tan, Shanghai Lung Tumor Clinical Medical Center, Shanghai Chest Hospital, Shanghai Jiao Tong University, Shanghai 200030, China.

E-mail: dr_tanqiang@sina.cn

Table S1. Data sources used in this study

Cohort	Data	Source
TCGA-LUAD	Clinical	https://api.gdc.cancer.gov/data/1b5f413e-a8d1-4d10-92eb-7c4ae739ed81
TCGA-LUAD	Counts	https://portal.gdc.cancer.gov/
TCGA-LUAD	FPKM	https://portal.gdc.cancer.gov/
TCGA-LUAD	Mutation	https://api.gdc.cancer.gov/data/1c8cfe5f-e52d-41ba-94da-f15ea1337efc
TCGA-LUAD	Aneuploidy score, fraction genome altered, MANTIS score, TMB	https://www.cbioportal.org/study/clinicalData?id=luad_tcga_pan_can_atlas_2018
TCGA-LUSC	Clinical	https://api.gdc.cancer.gov/data/1b5f413e-a8d1-4d10-92eb-7c4ae739ed81
TCGA-LUSC	FPKM	https://portal.gdc.cancer.gov/
GEO-NSCLC	GSE19188	https://www.ncbi.nlm.nih.gov/geo/query/acc.cgi?acc=GSE19188
GEO-LUAD	GSE72094	https://www.ncbi.nlm.nih.gov/geo/query/acc.cgi?acc=GSE72094
GEO-LUAD	GSE41271	https://www.ncbi.nlm.nih.gov/geo/query/acc.cgi?acc=GSE41271
GEO-NSCLC	GSE135222	https://www.ncbi.nlm.nih.gov/geo/query/acc.cgi?acc=GSE135222

Table S2. The gene sets utilized for immune signature score in this study

Signature	Genes	PMID
CD8 T effector cells	CD8A, GZMA, GZMB, IFNG, CXCL9, CXCL10, PRF1, TBX21	29443960
Immune Checkpoint	CD274, PDCD1LG2, CTLA4, PDCD1, LAG3, HAVCR2, TIGIT	29443960
IFNG signature	IDO1, CXCL10, CXCL9, HLA-DRA, STAT1, IFNG	28650338
T cell-inflamed GEP	CCL5, CD27, CD274, CD276, CD8A, CMKLR1, CXCL9, CXCR6, HLA-DQA1, HLA-DRB1, HLA-E, IDO1, LAG3, NKG7, PDCD1LG2, PSMB10, STAT1, TIGIT	28650338
MHC-Class-I	B2M, TAP1, TAP2, HLA-A, HLA-B, HLA-C	28052254

Table S3. GSEA results of KEGG data sets between high and low INMT groups in the TCGA-LUAD cohort.

Table S4. GSEA results of REACTOME data sets between high and low INMT groups in the TCGA-LUAD cohort.

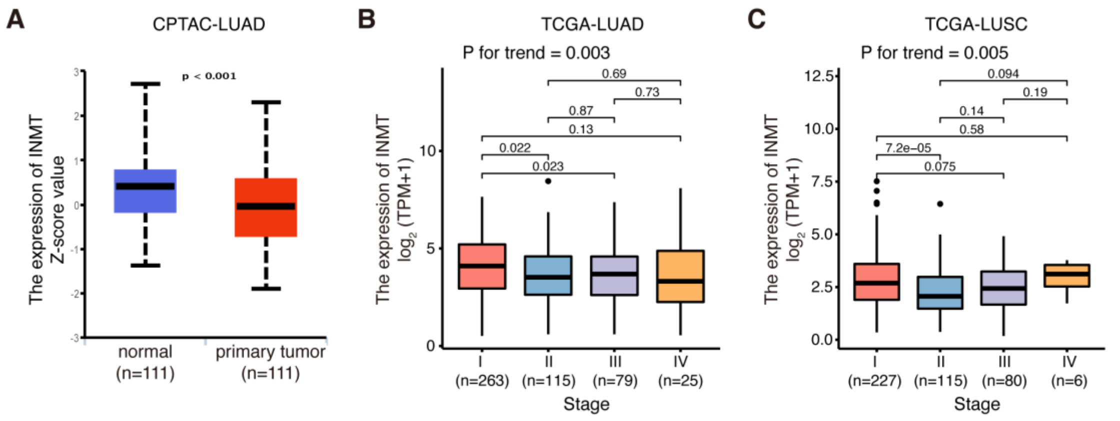

Figure S1. The expression of INMT in NSCLC.

(A) Protein expression of INMT in normal lung and LUAD tissues in CPTAC-LUAD dataset. (B and C) The expression of INMT in different pathological stages in TCGA-LUAD (B) and TCGA-LUSC (C) datasets. Spearman rank correlation test was applied to analyze the correlation of INMT expression with stage.

NSCLC, non-small cell lung cancer; LUAD, lung adenocarcinoma; LUSC, lung squamous cell carcinoma; TPM, transcripts per million mapped reads.

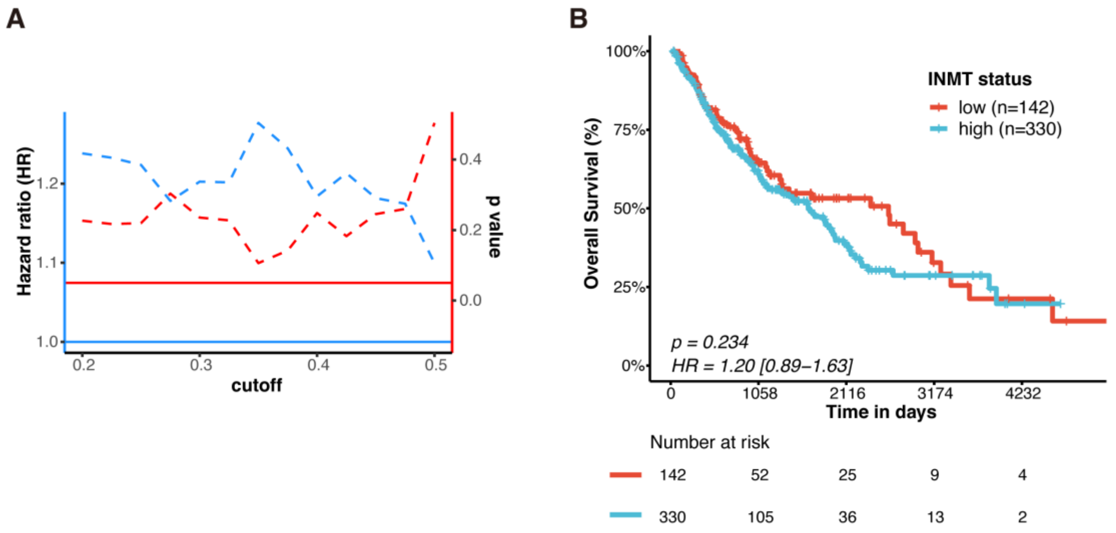

Figure S2. The analysis of the relationship of INMT expression and prognosis in LUSC patients.

(A) Hazard ratio (HR) and statistical results of INMT high-expression group versus low-expression group at different cutoffs in TCGA-LUSC cohort. The blue dashed line represents the HR value, the red dashed line represents the P value, and the solid red line represents P = 0.05. (B) Kaplan-Meier curve analysis of the prognostic significance of a high and a low expression of INMT in TCGA-LUSC cohort. The cutoff of 30% quantile was used to divide patients into low and high expression groups.

LUSC, lung squamous cell carcinoma; HR, Hazard ratio.
